# Probabilistic principal component analysis for metabolomic data

**DOI:** 10.1186/1471-2105-11-571

**Published:** 2010-11-23

**Authors:** Gift Nyamundanda, Lorraine Brennan, Isobel Claire Gormley

**Affiliations:** 1 School of Mathematical Sciences, University College Dublin, Ireland; 2 School of Agriculture, Food Science and Veterinary Medicine, Conway Institute, University College Dublin, Ireland

## Abstract

**Background:**

Data from metabolomic studies are typically complex and high-dimensional. Principal component analysis (PCA) is currently the most widely used statistical technique for analyzing metabolomic data. However, PCA is limited by the fact that it is not based on a statistical model.

**Results:**

Here, probabilistic principal component analysis (PPCA) which addresses some of the limitations of PCA, is reviewed and extended. A novel extension of PPCA, called probabilistic principal component and covariates analysis (PPCCA), is introduced which provides a flexible approach to jointly model metabolomic data and additional covariate information. The use of a mixture of PPCA models for discovering the number of inherent groups in metabolomic data is demonstrated. The jackknife technique is employed to construct confidence intervals for estimated model parameters throughout. The optimal number of principal components is determined through the use of the Bayesian Information Criterion model selection tool, which is modified to address the high dimensionality of the data.

**Conclusions:**

The methods presented are illustrated through an application to metabolomic data sets. Jointly modeling metabolomic data and covariates was successfully achieved and has the potential to provide deeper insight to the underlying data structure. Examination of confidence intervals for the model parameters, such as loadings, allows for principled and clear interpretation of the underlying data structure. A software package called MetabolAnalyze, freely available through the **R **statistical software, has been developed to facilitate implementation of the presented methods in the metabolomics field.

## Background

Metabolomics is the term used to describe the study of small molecules or metabolites present in biological samples. Examples of such metabolites include lipids, amino acids, bile acids, keto-acids. Studies of the concentration levels of these molecules in biological samples aim to enhance understanding of the effect of a particular stimulus or treatment [1-3]. The most commonly applied analytical technologies to metabolomic studies are nuclear magnetic resonance spectroscopy (NMR) [4] and mass spectrometry (MS) [5]. With respect to NMR-based metabolomics the data are usually in the form of spectra which are binned into regions of a specified width. Typically, the data generated by these methods are large and complex. Firstly, the number of observations n in metabolomics experiments is typically much less than the number of peaks (or variables) *p *in a spectrum, *n *≪ *p*. In such a situation, the application of standard parametric statistical methods such as regression is not straight forward as there are insufficient data for parameter estimation. Secondly, many metabolites may not have any relationship with the trait under study and they can induce variation which is not relevant, hampering comprehensive data analysis [6]. In view of these difficulties, when analyzing metabolomic data there is a genuine need for multivariate dimension reducing techniques which can take into account the complexities of the data and expose any underlying relationships. Principal components analysis (PCA) [7] is probably the most widely used technique for analyzing metabolomic data [8]. The popularity of PCA in metabolomics is due to the fact that it is a simple non-parametric method which can project the NMR or MS spectra into lower dimensional space, revealing inherent data structure, and providing a reduced dimensional representation of the original data. Despite its widespread use in metabolomics, PCA has several shortcomings. Most significantly, PCA does not have an associated probabilistic model, which makes assessing the fit of PCA to the data difficult and limits the potential to extend the scope of application of PCA. Additionally, PCA can fail to reveal underlying groups of subjects in the data, therefore providing a spurious view of the underlying data structure [9,10]. Other limitations include the inability of PCA to deal with missing data appropriately [11].

Such limitations can be addressed by deriving PCA from a probabilistic framework resulting in probabilistic PCA (PPCA) [12]. The merits of a probabilistic approach are manyfold. Firstly, the maximum likelihood solution of the PPCA model corresponds to PCA and hence the familiar characteristics (such as principal scores and loadings) of PCA are retained. With regard to model estimation, closed form solutions for parameter estimates exist. Additionally, the Expectation Maximization (EM) algorithm [13] can be employed to estimate the parameters of the PPCA model. The EM algorithm is computationally efficient and also has the capacity to deal with missing data [14]. The probability density based approach facilitates comparison of different PPCA models to determine the 'best' model for the data using statistically principled approaches. In practice, this allows selection of the number of required principal components in a statistically valid manner. Given the probabilistic footing of the PPCA model, the Bayesian inferential framework [15,16] can be employed for inference, facilitating the inclusion of any prior information the practitioner may have [17].

Perhaps the key advantage of approaching PCA from a probabilistic modeling point of view is the facility to assess the uncertainty associated with the resulting model output. In practice, this translates to the ability to examine the uncertainty in an observations' principal component score, or in the estimated loadings. In this article, the construction of confidence intervals for model parameters such as loadings using the jackknife method is illustrated. Thus a more principled and clear insight to the principal component solution is available under the probabilistic approach than under the traditional covariance matrix decomposition approach.

In general, metabolomics studies generate metabolomic data in addition to other phenotypic data, examples of which include age, gender and bmi (body mass index) in the case of human based studies. Inclusion of these covariates in multivariate models when analyzing metabolomic data is highly desirable in order to allow a comprehensive analysis of the data. In this article a novel extension of the PPCA model called probabilistic principal components and covariates analysis (PPCCA) is introduced which incorporates covariates into the model and facilitates joint modeling of metabolomic data and covariates. Another crucial benefit of the probability density based approach is that a collection of PPCA models can be combined to form a mixture of PPCA models (MPPCA) for nonlinear modeling purposes [18]. A mixture of PPCA models can be used to cluster subjects whilst facilitating dimensionality reduction of metabolomic data. This model is closely related to the mixture of factor analyzers used to cluster microarray expression data in [19]. The application of MPPCA analysis to metabolomic data is presented and highlights the danger of assuming a single underlying PPCA model in cases were (often unknown) groups of observations are present.

These statistical methods (PPCA, PPCCA and MPPCA) are illustrated through an application to two metabolomic datasets. A software package called MetabolAnalyze [20], freely available through the **R **statistical software [21], has been developed to facilitate implementation of the presented methods in the metabolomics community and elsewhere.

## Methods

Probabilistic PCA (PPCA) is a probabilistic formulation of PCA based on a Gaussian latent variable model and was first introduced by Tipping and Bishop in 1999 [12]. The PPCA model reduces the dimension of high-dimensional data by relating a *p*-dimensional observed data point to a corresponding *q*-dimensional latent variable through a linear transformation function, where *q *≪ *p*. Given the statistical model underpinning PPCA, extensions of the model are possible, and a wealth of statistical tools can be utilized. Such extensions and tools are detailed in what follows.

### Probabilistic Principal Components Analysis

Let x*_i _*= (*x_i_*_1_, . . . , *x*_*ip*_)*^T ^*be an observed set of variables (eg. an NMR spectrum) for observation *i *and ***u**_i _*= (*u*_*i*1_, . . . , *u_iq_*)*^T ^*be a latent variable corresponding to observation *i *in the latent, reduced dimension space. In terms of traditional PCA, u*_i _*can be viewed as the principal score of subject *i*. The PPCA model can be expressed as follows

$${\underline{x}}_{i}\;=\;W{\underline{u}}_{i}+\underline{\mu }+{\underline{\epsilon }}_{i}$$

where **W **is a *p × q *loadings matrix, μis a mean vector and ${\underline{\epsilon }}_{i}$ is multivariate Gaussian noise for observation *i*, i.e. *p*(${\underline{\epsilon }}_{i}$) = *MV N_p_*(0, σ^2^**I **) where **I **denotes the identity matrix. The latent variable u*_i _*is also assumed to be multivariate Gaussian distributed, *p*(u*_i_*) = *MV N_q_*(0, **I **). The conditional distribution of the observed data given the latent variable can then be expressed as

(1) $$p\left({\underline{x}}_{i}|{\underline{u}}_{i}\right)\;=\;MVN_{p}\left(W{\underline{u}}_{i}+\underline{\mu },\sigma^{2}I\right)$$.

The distribution of the observed data, *p*(*x_i_*), also known as the predictive distribution, can be derived from the convolution of *p*(*u_i_*) and *p*(*x_i_*|*u_i_*) giving

$$p\left({\underline{x}}_{i}\right)\;=\;MVN_{p}\left(\underline{\mu },WW^{T}+\sigma^{2}I\right)$$.

In contrast to the more conventional view of PCA which is a mapping from the high dimensional data space to a low dimensional latent space, the PPCA framework is based on a mapping from a latent space to the data space. The observed data *x_i _*is generated by first drawing a value for the latent variable u*_i _*from its unit variance multivariate Gaussian distribution, *p*(u*_i_*). The observed variable x*_i _*is then sampled, conditioning on the generated value for u*_i_*, from the isotropic distribution defined in (1).

Any observed data point x*_i _*can be represented in a latent space by its corresponding *q*-dimensional latent variable u*_i_*. The distribution of the latent variable given the observed data can be derived using Bayes' Theorem to give

(2) $$p\left({\underline{u}}_{i}|{\underline{x}}_{i}\right)=MVN_{q}\left(M^{-1}W^{T}\left({\underline{x}}_{i}-\underline{\mu }\right),\sigma^{2}M^{-1}\right)$$

where **M **is a *q × q *matrix defined as **M **= **W***^T ^***W **+ σ^2^**I**. A key benefit of the PPCA approach is that, not only is an estimate of the location of each observation in the lower dimensional space available through its expected value E(u*_i_*) = **M**^-1^**W***^T ^*(*x_i _*- μ), an estimate of its associated uncertainty is available through the covariance matrix *σ*^2^**M**^-1 ^in (2). This is in contrast to conventional PCA where the lower dimensional location (i.e. the score) of an observation is available, but the uncertainty associated with it is not. The parameters (**W**, μand *σ*^2^) of the PPCA model can be estimated using maximum likelihood. Maximizing the (log) likelihood function with respect to model parameters is non-trivial; in [12] it is demonstrated that the estimates do however have closed form solutions. Crucially, the log likelihood of the PPCA model is maximized when the columns of **W **span the principal subspace of conventional PCA [12]. Thus the maximum likelihood estimate (MLE) of the loadings matrix **Ŵ **in PPCA corresponds exactly to the loadings matrix in conventional PCA. Hence the model output in PPCA is exactly that obtained in conventional PCA, but with the additional advantages of uncertainty assessment and potential model extensions.

In this article maximum likelihood estimates of the model parameters are derived via the EM algorithm [13] because of its stability and widespread applicability. The EM algorithm is typically used to compute MLEs in probabilistic models when the model depends on unobserved variables or when some data are missing. The algorithm alternates between two steps until convergence: the expectation (E) step and the maximization (M) step. In the E-step, the expected values of the latent variables are estimated given the observed data and the current estimates of the model parameters. In the M-step, the model parameters are re-estimated by maximizing the log likelihood function using the expected values of the latent variables derived in the previous E-step. The two steps are repeated until convergence. Many convergence assessment criteria are available; some criteria are based on log likelihood gain between iterations while others use an estimate of the converged log likelihood value as a basis for stopping. Here Aitken's acceleration procedure [22] is used for convergence assessment. Specific details of the EM algorithm for the PPCA model are given in Additional File 1. An implementation of the algorithm is available in the package MetabolAnalyze through the **R **statistical software [21].

### Probabilistic Principal Components and Covariates Analysis

With its basis in a statistical model, the PPCA model can be extended in several ways. Given the availability and relevance of subject covariates in metabolomic studies, here the PPCA model is extended to facilitate joint modeling of covariates and metabolomic data, giving the probabilistic principal components and covariates analysis (PPCCA) model. This novel model extension is achieved by assuming that the latent variable distribution for observation *i *follows a multivariate Gaussian distribution centered at δ*_i _*rather than at the origin, i.e. *p*(u*_i_*) = *MV N_q _*(δ*_i _*, **I **), where

(3) $${\underline{\delta }}_{i}\;=\;\alpha {\underline{C}}_{i}=\left[\begin{array}{c} {\underline{\alpha }}_{1}^{T}\\ \vdots \\ \vdots \\ {\underline{\alpha }}_{q}^{T} \end{array}\right]\left[\begin{array}{c} 1\\ \vdots \\ c_{iL} \end{array}\right]$$

Here ***α*** is a *q *(*L *+ 1) matrix of parameters which capture the relationship between the latent variable and the covariates and C*_i _*is a (*L*+1) vector of an intercept term and the *L *covariates of observation *i*. The motivation behind this model extension is that a subject's covariates may influence their location in the principal subspace. Conditional on this location, the observed data point is then generated as in (1). Note that through the model definition (3) the covariates may have different effects on each of the dimensions of the principal subspace through the parameter vectors α_1_, . . ., α*_q_*.

Under the PPCCA model, the conditional distribution of x*_i _*given u*_i _*is the same as that of the PPCA model given in (1). The predictive distribution *p*(x*_i_*) differs from that of the PPCA model and is now defined as

$$p\left({\underline{x}}_{i}\right)=\;MVN_{p}\left(W{\underline{\delta }}_{i}+\underline{\mu },WW^{T}+\sigma^{2}I\right)$$.

The posterior distribution of the latent variable u*_i _*given the observed data x*_i _*is also affected by the inclusion of covariates and is defined to be

$$p\left({\underline{u}}_{i}|{\underline{x}}_{i}\right)\;=MVN_{q}\left(M^{-1}\left[W^{T}\left({\underline{x}}_{i}-\underline{\mu }\right)+\sigma^{2}{\underline{\delta }}_{i}\right],\sigma^{2}M^{-1}\right)$$.

The location (or score) of observation *i *in the latent space is given by E(u*_i_*) = **M**^-1^[**W***^T ^*(x*_i _*- μ) + *σ*^2^δ*_i_*], which depends on both the data point x*_i _*and the covariates of observation *i *through δ*_i_*. Thus when representing an observation in a reduced dimensional space the PPCCA model takes account of both the spectra data and the associated covariates. Deeper insight to the true underlying structure of the data is then feasible as possibly influential external factors are explicitly modeled.

The effect of covariates on the *q*th latent dimension can be explored through examination of the estimated regression parameter vector α*_q _*= (*α*_*q*0_, . . ., *α_qL_*)*^T ^*; these parameters can be interpreted within the context of the problem to provide insight to the type and strength of influence some covariates may have on the (often interpretable) latent dimensions.

Parameter estimation for the PPCCA model can be achieved via an efficient EM algorithm; Specific details of the EM algorithm for the PPCCA model are given in Additional File 1. An implementation of the algorithm is available in the package MetabolAnalyze through the **R **statistical software [21].

### Mixtures of Probabilistic Principal Components Analysis Models

The models discussed so far assume that the association between the observed data and the latent variable is linear. This assumption can be inadequate in a situation where the observations in the data set have an underlying group structure. In such cases the linearity assumption may not reveal all of the internal structures of the data [23]. Standard PCA also suffers from this phenomenon.

In many high throughput technologies which result in high dimensional data, interest often lies in identifying underlying sub groups within a set of observations. Exploring high dimensional data with underlying nonlinear structures therefore requires modeling attention. Employing a single PPCA model to model such data is not adequate, since PPCA provides a globally linear projection of the data. A collection of single PPCA models can be combined to obtain a mixture of probabilistic principal components analysis models (MPPCA) [18] which clusters observations into groups and reduces data dimension.

Under a MPPCA model, with probability *π_g_*, observation *i *is modeled as

$${\underline{x}}_{i}\;=\;W_{g}{\underline{u}}_{ig}+{\underline{\mu }}_{g}+{\underline{\epsilon }}_{ig}.$$

Here **W***_g _*and μ*_g _*are a *p *× *q *loadings matrix and the mean respectively for group *g*, and ${\underline{\epsilon }}_{ig}$ is a multivariate Gaussian noise process for observation *i*, given that *i *is a member of group *g*. The latent location for observation *i*, given that *i *is a member of group *g*, is denoted u*_ig_*. That is, with probability *π_g_*, observation *i *is modeled using a PPCA model with group Specific parameters.

Observation *i *is assumed to have been drawn from a finite mixture distribution with *G *components (or groups), i.e.

$$p({\underline{x}}_{i})\;=\;\sum_{g=1}^{G}\pi_{g}p({\underline{x}}_{i}|{\underline{\mu }}_{g},\Sigma_{g})$$

where *p*(x*_i_*| μ*_g_*, **Σ***_g_*) is a PPCA model for group *g *with mean parameter μ_*g *_and covariance matrix $\Sigma_{g}=W_{g}W_{g}^{T}+\sigma^{2}I$. The mixing proportion *π_g _*denotes the probability of an observation belonging to group $g\left(0\leq \pi_{g}\leq 1\;\text{and}\sum_{g=1}^{G}\pi_{g}=1\right)$. Note that for reasons of parsimony the error covariance ^2 ^has been constrained to be equal for all groups [24].

The MPPCA model can be fitted using a two stage EM algorithm called the Alternating Expectation Conditional Maximization (AECM) algorithm [25]. For clarity, the details of the AECM algorithm for the MPPCA model are deferred to Additional File 1. Under the MPPCA model, in addition to the latent location variable, the unobserved group membership of each observation is also viewed as a latent variable. Specifically, for each observation, a latent binary vector z*_i _*= (*z*_*i*1_, . . ., *z_iG_*)*^T ^*is imputed where *z_ig _*= 1 if observation *i *belongs to group *g *and 0 otherwise. At convergence of the AECM algorithm the estimate ${\hat{z}}_{ig}$ is the posterior probability of observation *i *belonging to group *g*. The MPPCA model clusters observations into groups by assigning them to the group to which they have highest posterior probability of membership. Thus clustering of observations and dimension reduction, through the use of principal components, are achieved simultaneously.

### Model Selection

A crucial advantage of working within a probabilistic framework is that a wealth of statistically based model selection tools can be utilized. This allows the determination of the "best" statistical model for the data, i.e. the optimal number of principal components *q *to retain and, in the case of the MPPCA model, the optimal value of *G*. Such choices are made on the basis of statistical principles instead of using traditional ad-hoc approaches, such as a scree plot.

The Bayesian Information Criterion (BIC) [26] is a popular model selection tool. The BIC is defined to be

(4) BIC = 2l−Kln(n)

where *l *is the maximum log likelihood value, *K *is the number of free parameters in the model and *n *is the number of observations. The model which yields highest BIC value is deemed the optimal model.

The BIC can be viewed as a criterion that rewards model fit (through the first term in (4)) but penalizes model complexity (through the second term in (4)). The penalization in the BIC is much stronger than that of the widely used Akaike Information Criterion [27] and typically selects more parsimonious models. Within the context of mixture models, the BIC has been widely employed, see [10,28,29].

Despite the tendency for the BIC to select parsimonious models, in high dimensional data settings it often exhibits noisy behaviour-the BIC can be undefined or perform poorly due to the occurrence of singularities for some starting values of the EM algorithm, for some models (i.e. different values of *q*), or for some numbers of groups (in the case of the MPPCA model). Additionally, diagnosing convergence of the EM algorithm in highly-parameterized models can be difficult, leading to noisy BIC values.

To eradicate this issue, here a regularized version of the BIC [30,31] is employed as a model selection tool. This modified version of the BIC evaluates the likelihood at the maximum a posteriori (MAP) estimator instead of the MLE. The MAP estimator is derived within the EM algorithm framework where a conjugate prior is included, and the convolution of the likelihood and prior are maximized at the M step. Here, a conjugate inverse gamma prior on *σ*^2 ^is employed throughout-the reported BIC values are based on the MAP estimate for *σ*^2 ^rather than on the MLE. Further details are provided in Additional File 1. This approach avoids singularities, and performs similarly to the BIC when such issues are absent. It also has the effect of smoothing noisy behavior of the BIC, which is often observed when parameter estimation is unstable.

### Jackknife resampling

The loadings of any probabilistic principal components based model can be used to identify variables responsible for the structure in the data. Rather than examining the (typically large number of) point estimates of the loadings alone, a gauge of the uncertainty associated with the loadings can be obtained through estimation of their standard errors.

Here the jackknife resampling method [32,33] is implemented. Standard errors are estimated by recomputing the loadings (i.e. re fitting the relevant PPCA model) with the *i*th observation removed from the dataset giving the loadings matrix **W**^-*i*^, for *i *= 1, . . ., *n*. The jackknife standard error for the *j*th loading on the *k*th principal component is then estimated as

$$SE\left({\hat{w}}_{jk}\right)\;=\;\sqrt{\frac{n-1}{n}\sum_{i=1}^{n}{\left[w_{jk}^{-i}-{\bar{w}}_{jk}\right]}^{2}}$$

Where $\bar{W}=\frac{1}{n}\sum_{i=1}^{n}W^{-i}$.

The standard errors can then be used to compute 95% confidence intervals (CIs) for the individual loadings. Such CIs can be used to identify loadings which differ significantly from zero on a selected principal component in the optimal model. Those variables whose loadings are significantly different from zero relate to the variables responsible for the structure within the data. This approach therefore provides a sparse list of relevant variables.

Computation time is often an issue when using the EM algorithm to fit statistical models, and employing the jackknife technique to obtain standard errors would clearly exacerbate this problem. In practice computation times are considerably reduced by choosing good starting values for the algorithm for each of the *n *runs when using the jackknife. Here the maximum likelihood estimates of the model parameters when fitted to the entire data set are employed as starting values for each of the jackknife runs, considerably reducing computational costs.

### Metabolomic datasets

The datasets used here were derived from a study previously reported [34]. Brie y, animals were randomly assigned to two treatments groups and treated with pentylenetetrazole (PTZ, treated group) or saline (control group) for a period of 4 weeks. A third treatment group consisted of animals who received one injection only and these data are not used within this paper. Throughout the treatment period urine was collected from the animals in collection tubes containing 1% sodium azide surrounded by ice. The animals had no access to food during this time but had free access to water. At the end of the treatment period brain regions were isolated and metabolites extracted as previously described [34]. NMR spectra were acquired and the spectra were integrated into bin regions of 0.04 ppm using AMIX (Bruker) excluding the water regions (4.0-6.0 ppm).

The urine dataset used herein was constructed from NMR spectra acquired from urine collected on day ten of the study; it consists of 18 spectral profiles (from 9 treated and 9 control animals) over 189 spectral bin regions.

The brain dataset comes from animals in the control group only with spectra acquired from tissues from four brain regions: the pre-frontal cortex, hippocampus, cerebellum and brainstem. In total, there are 33 spectral profiles over 164 spectral bin regions.

## Results

### Application of PPCA to metabolomic data

To explore the effect of treatment with PTZ, a PPCA model was fitted to the urinary metabolomic data. Parameter estimation was achieved via the EM algorithm. A number of PPCA models with varying numbers of principal components was fitted; a modified Bayesian Information Criterion (BIC) was used to aid selection of the optimal model (that is, the required number of principal components *q*) where a higher value of the criterion indicates a preferable model.

**Figure 1:**
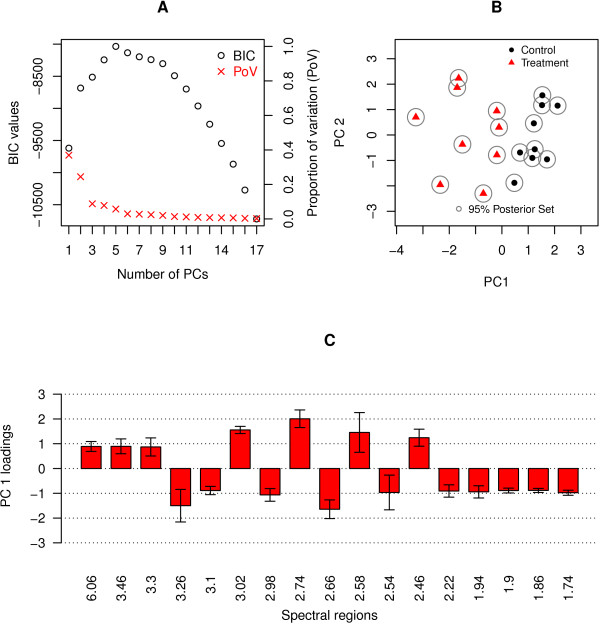
**Results of fitting a PPCA model to the urine dataset**. **A**. Plot of the modified BIC values and the proportion of variation explained by each model: the higher the BIC value the better the model. **B**. The scores plot: the red triangles denote the subjects in the treatment group and the black dots denote those in the control group. The grey ellipses are the 95% posterior sets indicating the uncertainty associated with each estimated score. **C**. A barplot of spectral bins with loadings which are significantly different from zero and greater in absolute value than 0.8. The barplot shows how the selected spectral regions load on PC 1 and their corresponding 95% confidence intervals.

The fitted PPCA model is illustrated in Figure 1. Figure 1A shows that the modified BIC is maximized by a model with five principal components (PCs); such a model explains 84% of the variation within the urine spectra data. Should the principal scores be required as a reduced dimensional input to further statistical modeling of the data, the modified BIC clearly indicates that a five dimensional representation is optimal. Overall, it represents an unambiguous means of selecting the optimal number of principal components. For clarity, the scores and loadings on the first two principal dimensions are illustrated. The scores plot (Figure 1B) reveals that grouping of animals with respect to their treatment status is evident on the first principal component. The 95% posterior sets are small, indicating little uncertainty in the scores. The associated loadings (which in turn correspond to metabolites) are presented in Additional file 2.

The 95% confidence intervals (CIs) of the individual loadings are estimated using the jackknife technique -- these CIs are used to identify loadings which are significantly different from zero. Of the 189 spectral bins in the urine spectra dataset, 86 have loadings on PC 1 significantly different from zero.

In order to further identify metabolites that strongly influence the separation of the treatment and control groups and which will serve as markers for treatment response, significant loadings greater (in absolute value) than 0.8 were selected. The cutoff value of 0.8 was chosen by examining a frequency plot of the (absolute) loading values of the significant spectral bins. A region in the plot in which the number of selected significant spectral bins drops steeply while the loading values remain relatively constant may be used as an indication of a cutoff point. In the current analysis, the plot (in Additional file 2) drops at the value 0.8.

Seventeen spectral bins had (absolute) loading values greater than the cutoff point of 0.8 and are illustrated in Figure 1C, along with their 95% CIs. Further analysis was performed to identify which of these bin regions differ significantly between the two treatment groups (using a t-test, correcting for multiple testing). Of the seventeen spectral bins, ten had signal intensities which were significantly different between the two groups. Included in these changes were bin regions due to the drug administered (1.74 ppm 1.86 ppm, 1.9 ppm, 2.22 ppm, and 3.1 ppm). Taurine levels (3.30 ppm and 3.46 ppm), dimethylamine (2.74 ppm) and one unassigned peak (6.06 ppm) were significantly lower in the treated group, while isocitrate levels were significantly higher (2.98 ppm) in the treated group.

### Application of PPCCA to metabolomic data

In addition to the urine metabolomic data the weight of each of the eighteen animals was recorded. Inclusion of this covariate in the analysis was achieved using the PPCCA model. Specifically, the covariate is incorporated to the PPCA model by allowing it to influence the score of each animal in the principal subspace. From (3), the expected value of the score of animal *i*, δ*_i_*, is modeled as a linear function of its covariate (i.e. weight):

$${\underline{\delta }}_{i}=\alpha {\underline{C}}_{i}=\left[\begin{array}{cc} \alpha_{10} & \alpha_{11}\\ \vdots & \vdots \\ \alpha_{q0} & \alpha_{q1} \end{array}\right]\left[\begin{array}{c} 1\\ c_{i1} \end{array}\right]$$

where *α*_10_, . . ., *α*_*q*0 _are intercept parameters for each dimension of the principal subspace and *α*_11_, . . ., *α*_*q*1 _are slope parameters for the weight covariate (denoted *c*_*i*1 _for animal *i*) for each dimension of the principal subspace. Hence the influence of an animal's weight on their score in the principal subspace is explicitly modeled and can be interpreted through the parameter matrix *α*.

**Figure 2:**
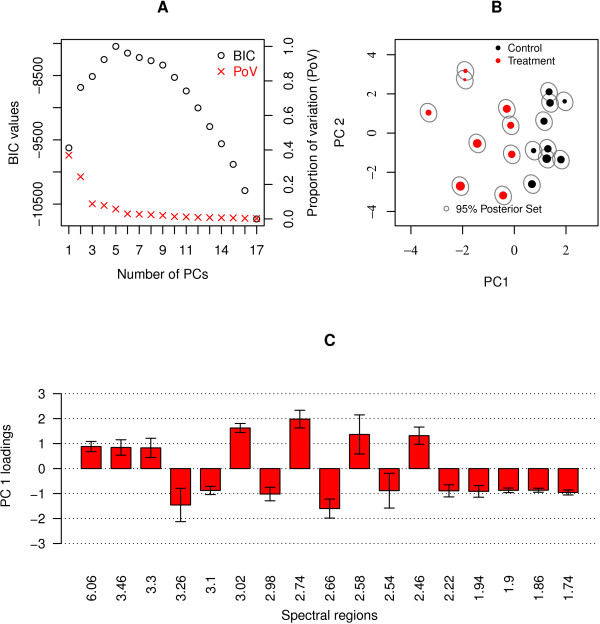
**Results of fitting a PPCCA model to the urine dataset with weight as a covariate**. **A**. Plot of the modified BIC values and the proportion of variation explained by each model: the higher the BIC value the better the model. **B**. The scores plot: the red dots denote the subjects in the treatment group and the black dots denote those in the control group. Dot size reflects a subject's weight. (Larger dots suggest heavier subjects.) The grey ellipses illustrate 95% posterior sets indicating the uncertainty associated with each score. **C**. A barplot of spectral bins with loadings which are significantly different from zero and greater in absolute value than 0.8. The barplot shows how the selected spectral regions load on PC 1 and their corresponding 95% confidence intervals.

The EM algorithm was employed to fit the PPCCA model to the urine spectra and the weight covariate. Figure 2A shows that the modified BIC is maximized by a model with five principal components (PCs). Examination of the (two dimensional for clarity) scores plot (Figure 2B) and the loadings plot (in Additional file 2) indicates that while the parameter estimates on the first principal component dimension remain relatively unchanged from the fitted PPCA model, the estimates on the second principal component dimension differ slightly. The general structure of the scores and loadings remains relatively unchanged suggesting that the animal's weights are not influencing the separation between treated and control animals on PC1. Additionally, it is apparent (Figure 2B) that the uncertainty associated with the estimated scores increases under the fitted PPCCA model. Selection of inferential bin regions identified the same seventeen regions as those obtained using the PPCA model (Figure 2C).

The influence of the weight covariate can be quantified by examining the associated regression parameter matrix, detailed in Table 1. Standard errors of the PPCCA regression parameters were estimated using jackknife resampling. The parameter estimates and the associated 95% CIs show that an animal's weight has a significant negative effect on the second principal component only (*α*_20 _= 4.08 and *α*_21 _= -6.28) and is not contributing to the treatment effect observed on PC1.

**Table 1:** Regression parameter estimates from the fitted PPCCA model.

	Intercept	Slope
PC 1 (α_1_)	-0.87 (-3.34, 1.61)	1.34 (-2.47, 5.15)
PC 2 (α_2_)	**4.08 **(2.44, 5.72)	**-6.28 **(-8.57, -3.99)
PC 3 (α_3_)	0.86 (-0.07, 1.78)	-1.32 (-2.77, 0.14)
PC 4 (α_4_)	0.18 (-1.03, 1.38)	-0.28 (-2.13, 1.58)
PC 5 (α_5_)	0.03 (-2.20, 2.26)	-0.04 (-3.32, 3.23)

95% CIs are given in parentheses. Those estimates significantly different from zero are highlighted in bold.

### Application of MPPCA to metabolomic data

MPPCA was applied to the brain metabolomic data in order to determine the number of inherent groups (if any) in the data. This application also illustrates the pitfalls of assuming a single PPCA model when exploring heterogeneous data.

**Figure 3:**
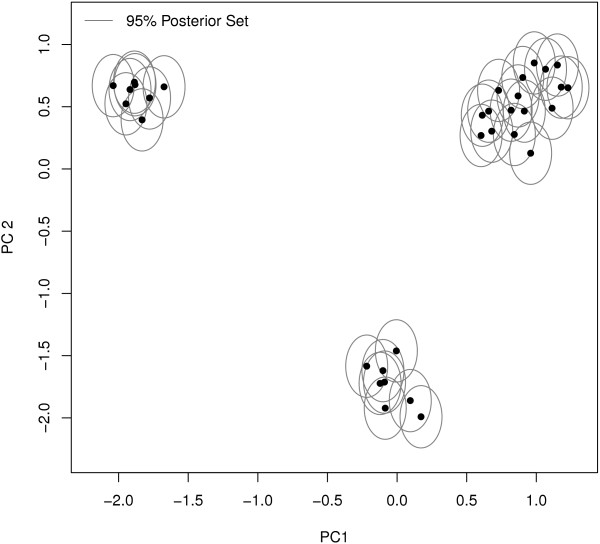
**The scores plot for a single PPCA model fitted to the brain spectra**. Each black dot denotes a score in the two dimensional principal subspace. The grey ellipses are the 95% posterior sets illustrating the uncertainty associated with each score. An underlying group structure is clearly apparent.

The scores plot resulting from fitting a single PPCA model to the brain spectral data is illustrated in Figure 3-it is immediately clear that there is a grouping (or clustering) structure within the set of 33 observations. With such a strong clustering structure it would seem extremely unlikely that the same set of principal axes would be relevant to each group. Fitting a MPPCA model simultaneously clusters the data into groups and reduces the dimension of the data within each group.

**Figure 4:**
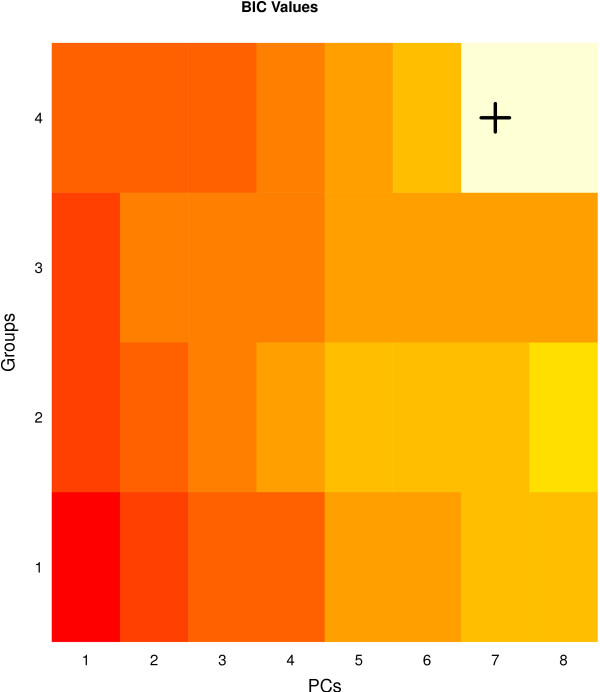
**A heatmap of the BIC values for MPPCA models fitted to the brain spectra data**. Colour represents the BIC value for each model -- the lighter the color the higher the BIC value and the better the model. The optimal model with *G *= 4 and *q *= 7 is indicated with a cross.

Thirty-two different MPPCA models were fitted to the 33 spectra by varying the number *G *of PPCA models in the mixture (each representing a group) from 1 to 4 and the number of principal components *q *from 1 to 8. Figure 4 is a heat map illustrating the modified BIC value for each fitted MPPCA model; the BIC suggests that the optimal model is the MPPCA model with four groups and seven principal components. This model can be used to cluster the observations into four groups and to visualize the data in each group within its principal subspace, hence exploring the structure relevant to each group. This method provides an objective means of identifying groups within the data.

In this illustrative example of the clustering and dimension reducing ability of the MPPCA model, the origin of each of the spectra was known. Thus, treating the brain region of origin as an observations' 'true' group, the clustering performance of the method can be assessed, where each observation is assigned to the group for which they have largest posterior probability of membership, under the optimal MPPCA model. In the current example, the model correctly clusters all observations into their brain group of origin (Table 2). Furthermore, the model correctly separates the prefrontal cortex and hippocampus samples which overlap in the scores plot under the PPCA model (Figure 3).

**Table 2:** Cross tabulation of the group membership of subjects based on the estimated MPPCA model and the brain region of origin.

	Cerebellum	Brain stem	Pre-frontal cortex	Hippocampus
Group 1	8	0	0	0
Group 2	0	8	0	0
Group 3	0	0	9	0
Group 4	0	0	0	8

The usefulness of this approach in the metabolomics field lies in its application to studies where the number of underlying groups and the group membership of each subject is unknown-the MPPCA model can be used to identify *G *and the members of each group within a study. Examples of such studies include the identification of disease phenotypes or treatment responsive phenotypes.

## Conclusions

Principal components analysis is the dominant statistical method currently employed within the field of metabolomics. Principal components analysis has many merits and is particularly well used and understood by metabolomic researchers. However, the scope of principal components analysis is limited and extensions (to make use of additional data sources, for example) are not possible, due to the lack of an underlying statistical model. As metabolomic research becomes more prevalent and data intensive, the development of methods which retain the familiar characteristics of principal components analysis while having additional analytical properties is of immediate importance.

This article demonstrates how probability density based methods can be used in the analysis of data resulting from metabolomics studies. Probabilistic principal components analysis (PPCA), and its equivalence with traditional PCA, is introduced in [12]. Thus PPCA retains the familiar and useful properties of PCA, but is based on a flexible statistical model. Standard statistical tools are then available for use -- in this article a model selection criterion is employed as a principled approach to selecting the number of principal components to retain. Additionally, uncertainty in the model estimates is assessed and standard errors are derived through the use of the jackknife technique. This provision of standard errors further aids model interpretation as inference on important model parameters such as loadings can be performed. Here, standard errors are employed to construct confidence intervals which are then used to indicate which loadings (and in turn metabolites) underlie the data structure.

In this article a novel model extension for PPCA, principal components and covariates analysis (PPCCA), is proposed. The PPCCA model offers a flexible way of including informative additional information in the PPCA model. In the context of metabolomics, this of particular interest, as covariates can be hugely influential on the metabolomic pro le. Jointly modeling such data in conjunction with metabolomic data is essential to facilitate comprehensive data analysis and understand the true metabolic changes occurring as a result of a particular stimulus. Overall, incorporating covariates in the PPCA model directly models any variation due to the covariates, thus ensuring that the principal components provide a clear picture of the structure underlying the data.

The use of a mixture of PPCA models as a simultaneous clustering and dimension reduction technique for metabolomic data was demonstrated successfully. This application represents a robust approach to identifying the number of groups within a dataset. It has great potential use within the metabolomics field for identifying metabotypes which are responsive to certain treatments. Additionally, a mixture of probabilistic principal components and covariates analyzers is an intuitive model extension which would provide clustering, dimension reduction and covariate modeling capabilities.

Overall, the present study details novel methods for analysis of metabolomics data which are freely available through a software package called MetabolAnalyze [20]; the package provides the facility to fit a PPCA model, a PPCCA model or a MPPCA model to metabolomic data, or indeed any other suitable data set.

## Authors' contributions

LB was involved in the study hypothesis, data interpretation and manuscript writing. CG was involved in the study hypothesis, data interpretation and manuscript writing. NG was involved in implementation and manuscript writing. All authors read and approved the final manuscript.

## Authors information

Nyamundanda Gift is PhD candidate in the PhD in Bioinformatics and Systems Biology programme in University College Dublin. Dr. Lorraine Brennan is a lecturer in Nutritional Biochemistry in the School of Agriculture, Food Science and Veterinary Medicine, Conway Institute, University College Dublin. Dr. Isobel Claire Gormley is a lecturer in Statistics in the School of Mathematical Sciences, University College Dublin.

## Supplementary Material

Additional file 1 **Statistical details of model fitting**. Click here for file

Additional file 2 **Loadings plots and plots to aid selection of the number of influential spectral bins**. Click here for file
