# Supplementary material for: Probabilistic principal component analysis for metabolomic data
Source: BMC Bioinformatics. 2010 Nov 23;11:571. doi: 10.1186/1471-2105-11-571 (PMC3006395; doi:10.1186/1471-2105-11-571)
Supplement: Additional file 1 — Statistical details of model fitting. [file 1471-2105-11-571-S1.PDF]

Additional File 1:
Statistical details of model fitting.

1 Fitting the Probabilistic Principal Components and Covariates Analysis model via the EM algorithm.

The EM algorithm is an iterative algorithm which provides maximum likelihood estimates of model parameters by alternating between E (expectation) and M (maximization) steps. The EM algorithm is particularly useful in the presence of missing data. In the context of the PPCCA model, the latent location variables (or 'scores') $\underline{u}_i$ are treated as 'missing' data. Combining the observed data and the missing data for subject i gives the complete data $(\underline{x}_i, \underline{u}_i)$. Given that $p(\underline{x}_i|\underline{u}_i) = MVN_p(\mathbf{W}\underline{u}_i + \underline{\mu}, \sigma^2\mathbf{I})$ and $p(\underline{u}_i) = MVN_q(\underline{\delta}_i\ , \mathbf{I}\ )$, the corresponding complete data log likelihood function is

$$\ell_c = \sum_{i=1}^{n} \left[\ln MVN_p(\mathbf{W}\underline{u}_i + \underline{\mu}, \sigma^2\mathbf{I}) + \ln MVN(\underline{\delta}_i, \mathbf{I})\right].$$

Maximum likelihood estimates of the model parameters are obtained by maximizing ℓ_c via the E and M steps of the EM algorithm. In the E-step the expected value of the complete data log likelihood is computed, conditional on the observed data and all current parameter values i.e.

$$\begin{aligned}\mathbb{E}[\ell_c] &= A + \frac{np}{2}\ln(\sigma^{-2}) - \frac{1}{2}\sum_{i=1}^{n}\{\sigma^{-2}(\underline{x}_i - \underline{\mu})^T(\underline{x}_i - \underline{\mu}) \\ &\quad -2\sigma^{-2}(\underline{x}_i - \underline{\mu})^T\mathbf{W}\mathbb{E}[\underline{u}_i] + \sigma^{-2}tr(\mathbf{W}^T\mathbf{W}\mathbb{E}[\underline{u}_i\underline{u}_i^T]) \\ &\quad +\mathbb{E}[(\underline{u}_i - \underline{\delta}_i)^T(\underline{u}_i - \underline{\delta}_i)]\}\end{aligned}$$

where $A = \frac{n(p+q)}{2}\ln(2\pi)$. Hence, the E-step of the EM algorithm is completed by computing the conditional expectations:

$$\begin{aligned}\mathbb{E}[\underline{u}_i] &= \mathbf{M}^{-1}\mathbf{W}^T(\underline{x}_i - \underline{\mu}) + \sigma^2\mathbf{M}^{-1}\underline{\delta}_i \\ \mathbb{E}[\underline{u}_i\underline{u}_i^T] &= \sigma^2\mathbf{M}^{-1} + \mathbb{E}[\underline{u}_i]\,(\mathbb{E}[\underline{u}_i])^T\end{aligned}$$

where $\mathbf{M} = \mathbf{W}^T\mathbf{W} + \sigma^2\mathbf{I}$.

In the M-step of the EM algorithm the expected complete data log likelihood is maximized with respect to the model parameters, $\boldsymbol{\alpha}$, $\mathbf{W}$ and σ^2. The MLE $\hat{\underline{\mu}}$ is given by the mean of the data $\bar{\underline{x}}$. Maximizing $\mathbb{E}[\ell_c]$ with respect to $\boldsymbol{\alpha}$, $\mathbf{W}$ and σ^2 provides the estimates:

$$\hat{\boldsymbol{\alpha}} = \sum_{i=1}^{n}\mathbb{E}\left[\underline{u}_i\right]\underline{C}_i^T\left(\sum_{i=1}^{n}\underline{C}_i\underline{C}_i^T\right)^{-1}$$

$$\hat{\mathbf{W}} = \left[\sum_{i=1}^{n}(\underline{x}_i - \underline{\mu})(\mathbb{E}[\underline{u}_i])^T\right]\left[\sum_{i=1}^{n}\mathbb{E}[\underline{u}_i\underline{u}_i^T]\right]^{-1}$$

$$\hat{\sigma}^2 = \frac{1}{np}\left[ntr(S) - 2\sum_{i=1}^{n}(\underline{x}_i - \underline{\mu})^T \mathbf{W}\mathbb{E}[\underline{u}_i] + tr\left(\mathbf{W}^T\mathbf{W}\mathbb{E}[\underline{u}_i\underline{u}_i^T]\right)\right]$$

where $S = \frac{1}{n}\sum_{i=1}^{n}(\underline{x}_i - \underline{\mu})(\underline{x}_i - \underline{\mu})^T$ and $\underline{\delta}_i = \boldsymbol{\alpha}\underline{C}_i$ is as defined in (3). The EM algorithm alternates between the E and M-steps until convergence; on convergence, the estimates are the maximum likelihood estimates of the model parameters $\hat{\boldsymbol{\alpha}}, \hat{\mathbf{W}}, \hat{\sigma}^2$, and the estimated value of $\underline{u}_i$ is the latent score of subject i. Here algorithmic convergence is assessed by Aitken's acceleration criterion [1].

As the PPCA model is equivalent to the PPCCA model with $\boldsymbol{\alpha} = \mathbf{0}$, to fit the PPCA model the E and M-steps proceed as outlined above with $\boldsymbol{\alpha}$ constrained to $\mathbf{0}$.

The BIC model selection criterion displays noisy behaviour when used to select the optimal number of principal components for the PPCA model and the PPCCA model for high dimensional metabolomic data. This issue is eradicated by employing a modified version of the BIC where a conjugate prior for the error variance σ^2 is employed. Thus at the M-step of the EM algorithm the sum of the expected complete data log likelihood and the log prior is maximized with respect to σ^2, stabilizing the algorithm. The BIC is then evaluated at the MLEs $\hat{\boldsymbol{\alpha}}$ and $\hat{\mathbf{W}}$ and at the posterior mode σ^2_{MAP}. In practice an Inverse Gamma$(\nu/2, \zeta/2)$ prior on σ^2 is used leading to the posterior mode estimate:

$$\sigma^2_{MAP} = \frac{Np\hat{\sigma}^2 + \zeta}{Np + \nu + 2}.$$

The values of the hyperparameters (ν and ζ) are chosen based on prior knowledge and/or such that the obtained modified BIC values are similar to the actual BIC values when singularities and noisy behaviour are absent.

2 Fitting a mixture of PPCA models via the AECM algorithm.

The Alternating Expectation Conditional Maximization (AECM) algorithm is a variant of the EM algorithm which has two cycles. Each cycle consists of an E-step and an M-step. The data specified as "missing" differs at each cycle. Similarly, at each M-step the expected complete data log likelihood is maximized with respect to a different set of parameters. Hence the algorithm alternates between expectation steps and conditional maximization steps. Dividing the missing data and parameters in this way often provides a more feasible computational approach to deriving MLEs than using a standard EM algorithm.

In the MPPCA model, the missing data consist of both the latent locations $\underline{u}_{i1}, \ldots, \underline{u}_{iG}$ and the latent group membership indicator variables $\underline{z}_i$ for each subject i. When estimating the parameters of the MPPCA model via the AECM algorithm, the first cycle considers the latent group membership indicator variables $\underline{z}_i$ as missing and conditions on the current values of the latent locations $\underline{u}_{i1}, \ldots, \underline{u}_{iG}$. Hence in the first cycle the 'complete data' for subject i is specified to be $(\underline{x}_i, \underline{z}_i)$. In the E-step of the first cycle the expected complete data log likelihood $\mathbb{E}[\ell_c]$ is computed with respect to the distribution of $\underline{z}_i$ conditional on $\underline{x}_i, \underline{u}_{i1}, \ldots, \underline{u}_{iG}$ and all other current parameter values. The density of the complete data for subject i $(\underline{x}_i, \underline{z}_i)$ is then

$$\begin{aligned} p(\underline{x}_i \underline{z}_i) &= p(\underline{x}_i | \underline{z}_i) p(\underline{z}_i) \\ &= \prod_{g=1}^{G} [\pi_g p(\underline{x}_i | z_{ig} = 1)]^{z_{ig}} \end{aligned}$$

where $p(\underline{x}_i|z_{ig}=1) = MVN_p(\mu_g, \mathbf{W}_g\mathbf{W}_g^T + \sigma^2\mathbf{I})$ is a single PPCA model fitted to the spectra of subjects currently assigned to group g. Note that the variance parameter σ^2 is constrained to be equal across all groups for computational stability. The expected value of the complete data log likelihood with respect to the distribution of the missing data is then

$$\mathbb{E}[\ell_c] = \sum_{i=1}^{n}\sum_{g=1}^{G}\mathbb{E}[z_{ig}]\left[\ln\pi_g + \ln p(\underline{x}_i|z_{ig}=1)\right] \qquad (1)$$

Therefore the E-step of the first cycle of the AECM algorithm is completed by computing the expected value of the multinomial random variable z_{ig}:

$$\mathbb{E}[z_{ig}] = \tau_{ig} = \frac{\pi_g p(\underline{x}_i|z_{ig}=1)}{\sum_{g'=1}^{G}\pi_{g'}p(\underline{x}_i|z_{ig'}=1)}.$$

In the M-step of the first cycle the expected complete data log likelihood (1) is maximized with respect to $\underline{\mu}_g$ and π_g for $g = 1,\ldots,G$ giving the estimates

$$\hat{\underline{\mu}}_g = \frac{\sum_{i=1}^{n}\tau_{ig}\underline{x}_i}{n_g}$$
$$\hat{\pi}_g = \frac{n_g}{n}$$

where $n_g = \sum_{i=1}^{n}\tau_{ig}$. This completes the first cycle of the AECM algorithm.

In the second cycle both $\underline{z}_i$ and $\underline{u}_{i1},\ldots,\underline{u}_{iG}$ are specified as missing data for subject i. In the E-step of the second cycle the expected complete data log likelihood $\mathbb{E}[\ell_c]$ is computed with respect to the conditional distribution of $\underline{u}_{ig}$ and $\underline{z}_i$ for $i=1,\ldots,n$ and $g=1,\ldots,G$:

$$\begin{aligned}\mathbb{E}[\ell_c] &= B + \sum_{g=1}^{G}\left[\frac{n_g p}{2}\ln(\sigma^{-2}) - 0.5n_g\sigma^{-2}tr(\mathbf{S}_g) + \sigma^{-2}\sum_{i=1}^{n}\tau_{ig}(\underline{x}_i - \underline{\mu}_g)^T\mathbf{W}_g\mathbb{E}[\underline{u}_{ig}]\right.\\ &\quad\left. -0.5\sigma^{-2}\sum_{i=1}^{n}\tau_{ig}tr(\mathbf{W}_g^T\mathbf{W}_g\mathbb{E}[\underline{u}_{ig}\underline{u}_{ig}^T])\right]\end{aligned}$$

where $\mathbf{S}_g = \frac{\sum_{i=1}^{n}\tau_{ig}(\underline{x}_i-\underline{\mu}_g)(\underline{x}_i-\underline{\mu}_g)^T}{n_g}$ and B is a constant. The E-step of the second cycle is therefore completed by recomputing τ_{ig} along with $\mathbb{E}[\underline{u}_{ig}]$ and $\mathbb{E}[\underline{u}_{ig}\underline{u}_{ig}^T]$ for $i=1,\ldots,n$ and $g=1,\ldots,G$ which are given as

$$\mathbb{E}[\underline{u}_{ig}] = \mathbf{M}_g^{-1}\mathbf{W}_g^T(\underline{x}_i - \underline{\mu}_g)$$
$$\mathbb{E}[\underline{u}_{ig}\underline{u}_{ig}^T] = \sigma^2\mathbf{M}_g^{-1} + \mathbb{E}[\underline{u}_{ig}](\mathbb{E}[\underline{u}_{ig}])^T$$

where $\mathbf{M}_g = \mathbf{W}_g^T\mathbf{W}_g + \sigma^2\mathbf{I}$.

In the M-step of the second cycle the expected complete data log likelihood is maximized with respect to $\mathbf{W}_g$ for $g=1,\ldots,G$ and σ^2 to give

$$\hat{\mathbf{W}}_g = \mathbf{S}_g\mathbf{W}_g(\sigma^2\mathbf{I} + \mathbf{M}_g^{-1}\mathbf{W}_g^T\mathbf{S}_g\mathbf{W}_g)^{-1}$$
$$\hat{\sigma}^2 = \frac{1}{p}\sum_{g=1}^{G}\hat{\pi}_g\left[tr(\mathbf{S}_g) - tr\left(\mathbf{S}_g\mathbf{W}_g\mathbf{M}_g^{-1}\tilde{\mathbf{W}}_g^T\right)\right]$$

where $\tilde{\mathbf{W}}_g$ is the value of $\mathbf{W}_g$ after the second E-step but prior to computing $\hat{\mathbf{W}}_g$. This completes the second cycle.

The AECM algorithm alternates between the two cycles detailed above until convergence (here assessed by Aitken's acceleration criterion). At convergence the parameter estimates obtained are the maximum likelihood estimates of the MPPCA model and the estimated value of $\underline{u}_{ig}$ is the latent score of subject i, given that i is a member of group g. The estimated values of $\underline{z}_i = (z_{i1}, \ldots, z_{iG})$ at convergence are the posterior probabilities of subject i being a member of group g; typically each subject is classified to the group for which they have highest posterior probability of membership. Thus both clustering of subjects into groups and dimension reduction are simultaneously achieved.

For the MPPCA model, a modified version of the BIC is employed to select the optimal model, both in terms of the number of groups G and the number of principal components q. Similar to the PPCA and PPCCA cases detailed above, a conjugate prior for the error variance σ^2 is employed in the MPPCA model. Thus at the second M-step of the AECM algorithm the sum of the expected complete data log likelihood and the log prior is maximized with respect to σ^2. The BIC is then evaluated at the MLEs $\hat{\pi}_g$, $\underline{\hat{\mu}}_g$ and $\hat{\mathbf{W}}_g$ for $g = 1, \ldots, G$ and at the posterior mode σ^2_{MAP}. In practice an Inverse Gamma$(\nu/2, \zeta/2)$ prior on σ^2 is used leading to the posterior mode estimate:

$$\sigma^2_{MAP} = \frac{N(p\hat{\sigma}^2 + \zeta)}{Np + \nu + 2}.$$

The values of the hyperparameters (ν and ζ) are chosen based on prior knowledge and/or such that the obtained modified BIC values are similar to the actual BIC values when singularities and noisy behaviour are absent.

References

[1] McLachlan GJ, Krishnan T: *The EM algorithm and Extensions*. New York: Wiley 1997.
