# Supplementary material for: Probabilistic principal component analysis for metabolomic data
Source: BMC Bioinformatics. 2010 Nov 23;11:571. doi: 10.1186/1471-2105-11-571 (PMC3006395; doi:10.1186/1471-2105-11-571)
Supplement: Additional file 2 — Loadings plots and plots to aid selection of the number of influential spectral bins. [file 1471-2105-11-571-S2.PDF]

Additional File 2: Plots to aid selection of the number of influential spectral bins.

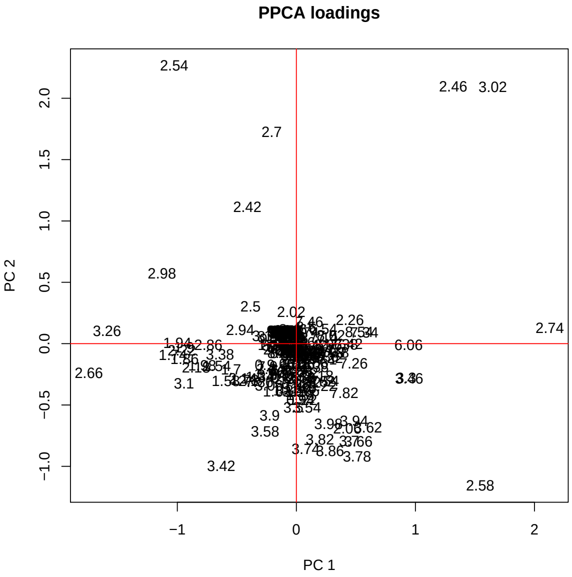

(a)

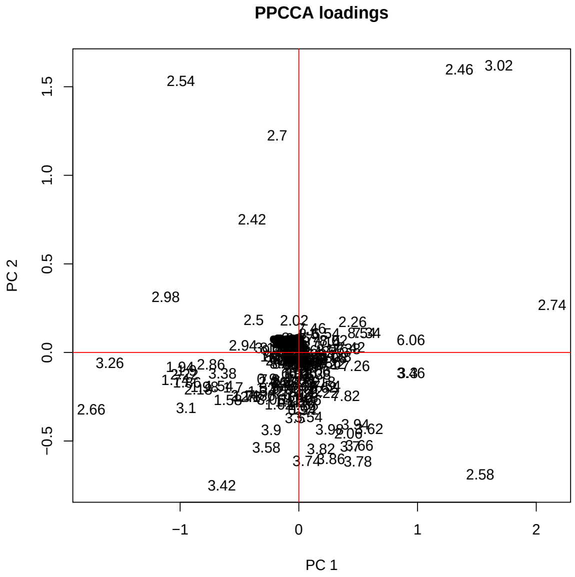

(b)

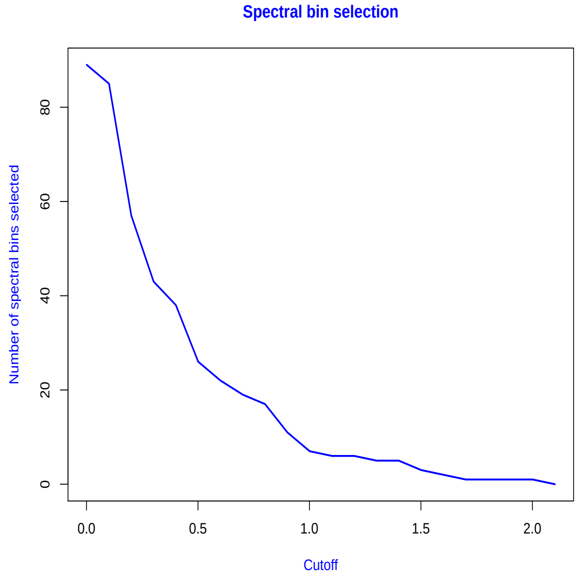

(c)

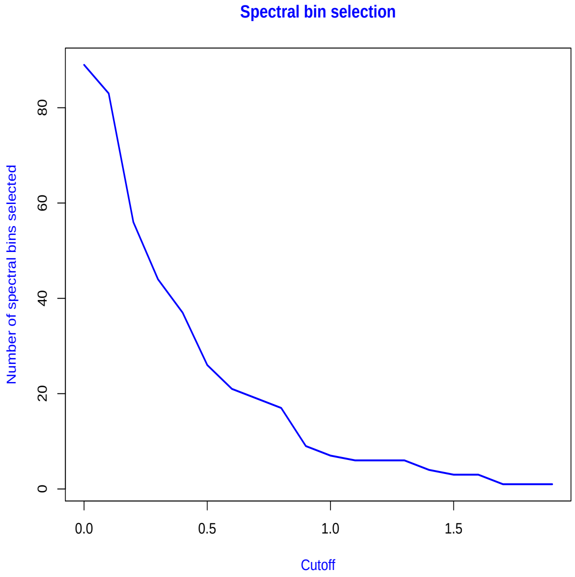

(d)

Figures (a) and (b) illustrate the resulting loading for each variable or spectral peak on the first two principal components under (a) the PPCA model and (b) the PPCCA model. Numbers denote the chemical shift values (0.04 ppm spectral bins.) Figures (c) and (d) are frequency plots of the (absolute) loadings values of spectral bins whose loadings are significantly different from zero. These plots are used to aid selection of a cutoff point to indicate influential significant spectral bins under (c) the PPCA model and (d) the PPCCA model.
